# Exogenous melatonin in the treatment of pain: a systematic review and meta-analysis

**DOI:** 10.18632/oncotarget.21504

**Published:** 2017-10-05

**Authors:** Chaojuan Zhu, Yunyun Xu, Yonghong Duan, Wei Li, Li Zhang, Yang Huang, Wei Zhao, Yutong Wang, Junjie Li, Ting Feng, Xiaomei Li, Xuehui Hu, Wen Yin

**Affiliations:** ^1^ Department of Emergency Medicine, Xijing Hospital, The Fourth Military Medical University, Xi’an 710032, China; ^2^ Department of Nursing, Xijing Hospital, The Fourth Military Medical University, Xi’an 710032, China; ^3^ Department of Human Anatomy, Histology and Embryology, The Fourth Military Medical University, Xi’an 710032, China; ^4^ Faculty of Nursing, College of Medicine, Xi'an Jiaotong University, Xi’an 710049, China; ^5^ Department of Dermatology, Xijing Hospital, The Fourth Military Medical University, Xi’an 710032, China

**Keywords:** melatonin, pain, brain-derived neurotrophic factor, meta-analysis

## Abstract

Melatonin is an important hormone for regulating mammalian circadian biology and cellular homeostasis. Recent evidence has shown that melatonin exerts anti-nociception effects in both animals and humans. However, according to clinical trials, the anti-nociception effects of melatonin are still controversial. The aim of this meta-analysis was to investigate the anti-nociception effects of melatonin premedication. The primary outcome was the effects of melatonin on pain intensity. The secondary outcomes included the number of patients with analgesic requirements, total analgesic consumption, and brain-derived neurotrophic factor (BDNF) levels. In total, 19 studies were included in the current meta-analysis. The pooling data show that melatonin significantly decreased the pain intensity, as evidenced by the pain scores. Moreover, melatonin administration also reduced the proportion of patients with analgesic requirements and BDNF levels. However, the effects of melatonin on total analgesic consumption still require further confirmation. Collectively, the current meta-analysis supports the use of melatonin for anti-nociception.

## INTRODUCTION

As an important hormone, melatonin is an accepted antioxidant and anti-inflammatory agent [1–8]. Melatonin is reported to regulate circadian biology, cellular autophagy and endoplasmic reticulum homeostasis [9–11]. Moreover, its protective roles against obesity, diabetes, sepsis and fibrosis have been widely observed [11–15]. In particular, its administration significantly improved sleep quality, anxiety, and depression [16–18]. Furthermore, melatonin receptors have been identified in the spinal cord tissue [19, 20]. Thus, melatonin may exert anti-nociception effects.

Interestingly, pain perception, especially heat and cold pain tolerance, is observed to vary diurnally, which may result from variations in melatonin levels [21]. In particular, acute pain stimuli influence the salivary melatonin levels [22]. Reduced endogenous melatonin exacerbates nerve injury-induced neuropathic pain [23], whereas melatonin administration significantly attenuates sleep deprivation-induced neuropathic pain [24]. Recent experimental evidence has also shown that melatonin could significantly alleviate pain behaviors under other conditions [19, 25–27].

Melatonin does not exhibit toxicity at the doses used [28]; even high-dose intravenous melatonin (100 mg) does not induce significant adverse effects [29–32]. Although some clinical trials have reported the anti-nociception effects of melatonin [33–45], several other studies have shown that melatonin has no significant effects on pain [16, 30, 46, 47]. To determine whether exogenous melatonin exerts anti-nociception effects in the human population, we conducted this meta-analysis.

## MATERIALS AND METHODS

### Data sources and searches

**Figure 1:**
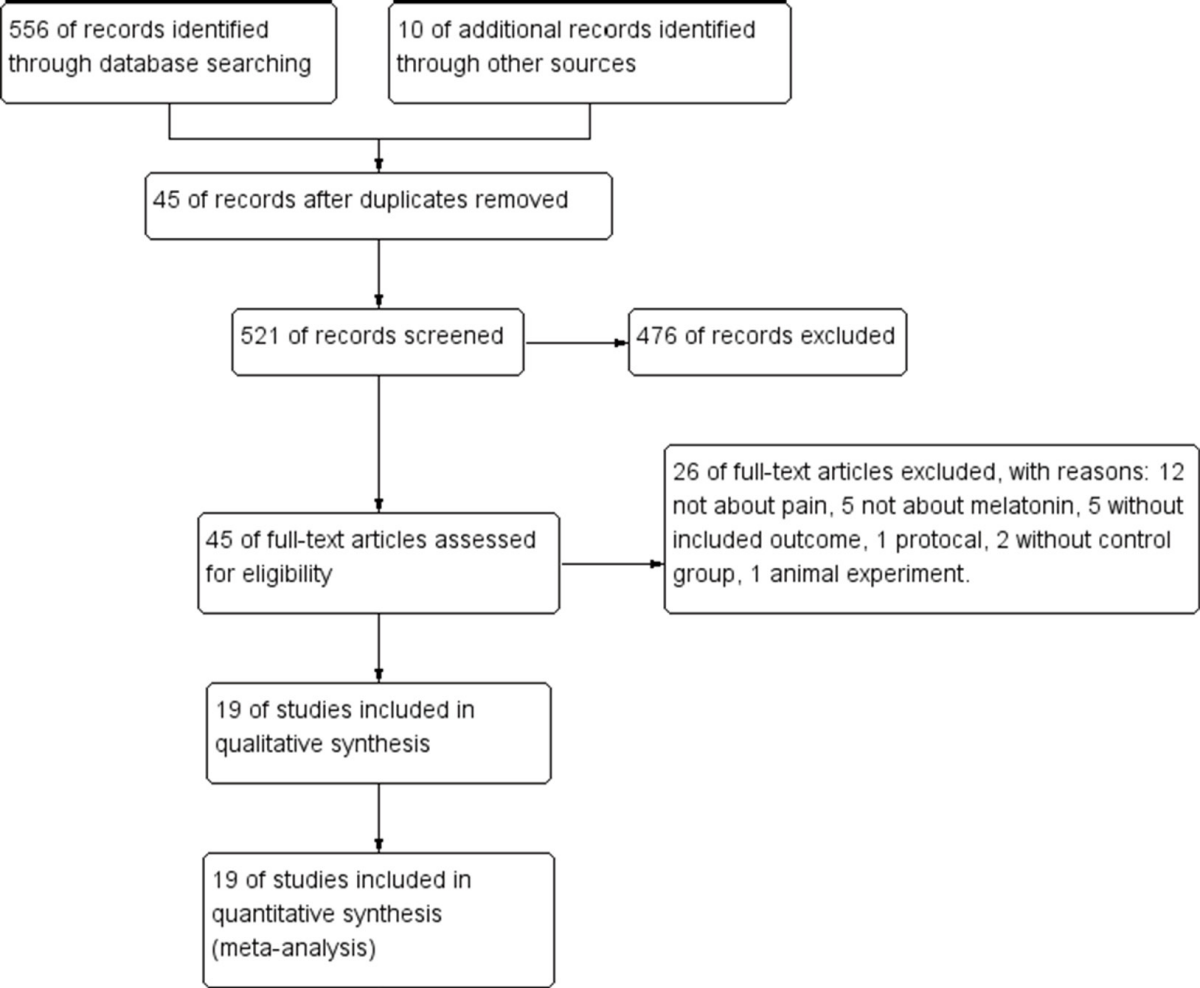
Study flow diagram VAS, Visual Analog Scale.

Two authors (Chaojuan Zhu and Yonghong Duan) independently searched the electronic databases, including MEDLINE (1990 to July 2017), EMBASE (1990 to July 2017), Scopus (1988 to July 2017), PsycINFO (1990 to July 2017), and the Cochrane Library (Issue 5 of 12, July 2017). Searches were limited to humans but were not limited by published language and publication type. References of published original articles, reviews and contacted experts were further searched. Melatonin, N-acetyl-5-methoxy tryptamine, pain, ache, dolor, pain management and analgesia were used as search terms. [((((pain) OR ache) OR dolor) OR pain management) and ((melatonin) OR N-acetyl-5-methoxy tryptamine)] were used as search phrases. The full study flow diagram is shown in Figure 1.

RCTs enrolled participants with any type of pain, including inflammatory pain, operation-associated pain, experimental pain and procedural pain. Melatonin could be administered orally or intravenously, either solely or in combination with other drugs. Both trials comparing melatonin with placebo or with positive control were included. Trial protocols without results, animal experiments, and studies without control groups were excluded.

### Data extraction

Two authors (Chaojuan Zhu and Xu Yunyun) independently extracted data, including study characteristics and the main outcomes. When discrepancies appeared, consensus was achieved through discussion with a third author (Yonghong Duan). Characteristics of patients, such as age, condition, number of patients and intervention, were collected.

The primary outcome was pain intensity, as evidenced by pain scores. Based on the results reported in RCTs, the Visual Analog Scale (VAS) was the most used scale in the included trials. Additionally, children’s anxiety and pain scales and premature infant pain profiles were also pooled. Secondary outcomes were the number of patients with analgesic requirements, total analgesic consumption, and brain-derived neurotrophic factor (BDNF) levels.

When only standard error (SE) was reported, SE was transformed to standard deviation (SD) using the formula: SD=SE×√n. When the 95% confidence interval (95% CI) was recorded, SD=√n×(upper limit-lower limit)/3.92. When only the median and interquartile range (IQR) were recorded, they were used to estimate the mean and SD: mean≈median, SD≈Norm IQR = (P75-P25)×0.7413 according to the Cochrane Handbook for Systematic Reviews of Interventions [48]. If the mentioned data were unavailable in the text, we attempted to contact the corresponding authors to obtain the related information. If the present data in the figures were not available in the text and it was impossible to retrieve them from the corresponding authors, ImageJ (National Institutes of Health, Bethesda, MD) was applied to measure the values in the figures.

### Quality assessment

Two authors (Chaojuan Zhu and Xu Yunyun) independently assessed the quality of the included trials according to the Jadad scales (5 items) [49]. Randomization, blinding, and withdrawals and dropouts were assessed. Table 1 presents the assessments of all included studies. The quality of the trial was described as high (score 5), moderate (score 4), or low (scores 1–3). The trials with low quality were not relied upon in the data pooling. Cohen’s kappa (κ) was utilized to measure the inter-rater agreement.

**Table 1:** Jadad scales of the included studies

References	Randomisation	Double blind	Withdrawals and dropouts	Total
Gitto 2016 [33]	2	2	1	5
Kirksey 2015 [46]	2	2	1	5
Marseglia 2015 [67]	2	2	1	5
Seet 2015 [47]	2	2	1	5
Andersen 2015 [30]	2	2	1	5
de Zanette 2014 [34]	2	2	1	5
Khezri 2013 [35]	2	2	1	5
Vidor 2013 [68]	2	2	1	5
Khezri 2013a [37]	2	2	1	5
Schwertner 2013 [38]	2	2	1	5
Stefani 2013 [36]	2	2	1	5
Gitto 2012 [39]	1	0	1	2
Hussain 2011 [40]	1	1	1	3
Borazan 2010 [41]	2	2	1	5
Ismail 2009 [42]	2	2	1	5
Caumo 2009 [44]	2	2	1	5
Mowafi 2008 [43]	2	2	1	5
Caumo 2007 [45]	2	2	1	5
Song 2005 [57]	1	2	1	4

### Statistical analysis

Review Manager analysis software (RevMan 5.2) was used to analyze the collected data according to the Cochrane Handbook for Systematic Reviews of Interventions [48]. For continuous data measured by different scales, we used weighted mean differences (WMDs) with applicable 95% CIs to measure the mean values or mean changes. WMDs were calculated for pain intensity, total analgesic consumption and BDNF levels. For dichotomous data, the odds ratios (ORs) with applicable 95% CIs were used for the pooling data. ORs were calculated for the number of patients with analgesic requirements.

**Figure 2:**
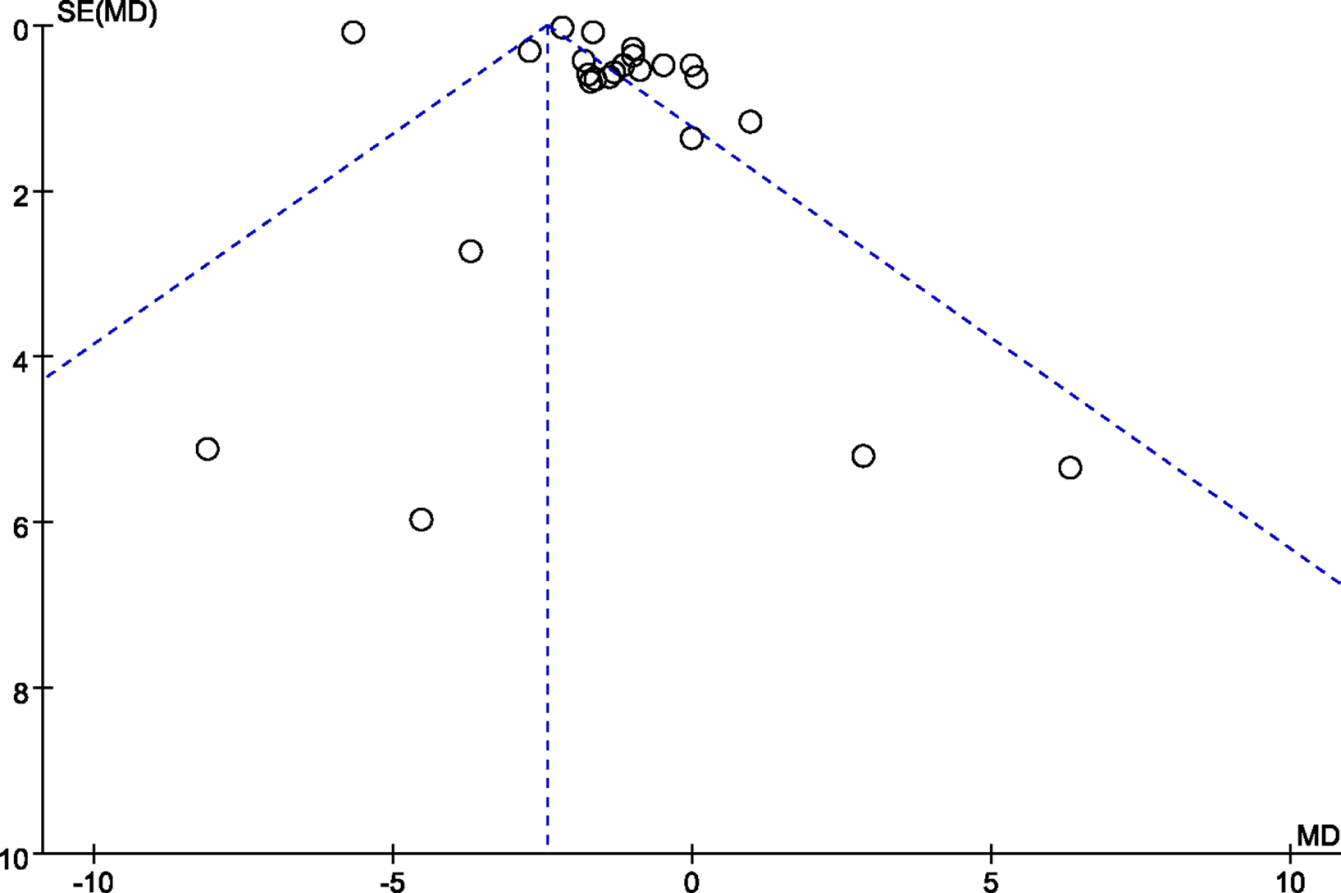
Funnel plot of comparison: pain score Eggers test: 1.121428, 95% CI: -3.588125 to 5.830981, *P* = 0.623.

I^2^ statistics were used to measure heterogeneity of the RCTs. If the I^2^ value was less than 50%, a fixed-effects model was applied. If the I^2^ value was 50% or more, a subgroup analysis was conducted. Subgroup analyses for pain intensity primarily included different pain types and compared groups, while analyses for total analgesic consumption included the stage used and compared groups. If a significant heterogeneity could not be identified by subgroup analysis, a random-effects model meta-analysis was used [48]. Sensitivity analyses were conducted to examine the stability of the pooling outcome and to trace the heterogeneity source by excluding the sole study with low quality or high risk of bias. Publication bias was tested using a funnel plot (Figure 2) and Egger’s test [50, 51].

## RESULTS

Figure 1 present the full study screening process. Ultimately, 19 studies were included in this meta-analysis. Their quality assessment is shown in Table 1. High inter-rater reliability of initial decisions on the inclusion of studies was indicated (κ = 0.838, 95% CI: 0.53–1). All of the included studies were RCTs. Among them, 18 studies were randomized double-blind trials, 14 studies were placebo-controlled trials, and 17 studies were assessed as high quality. In total, 586 patients were included in the melatonin groups, while 507 patients received control therapy, including placebo and standard therapy. The characteristics of these studies are summarized in Table 2.

**Table 2:** Study characteristics

	References	Population	Condition	Experiment group	Numbers	Control groups	Numbers	Administration
Operation-associated pain	Gitto 2016 [33]	Children aged 5 to 14	Elective surgery	Melatonin 0.5 mg/kg (max 20 mg)	46	Midazolam 0.5 mg/kg	46	Oral premedication 40 min before anaesthesia
	Kirksey 2015 [46]	ASA I-III patients aged 18 to 90	Total knee arthroplasty under regional anesthesia	Melatonin 5 mg	19	Placebo	18	Oral premedication at the bedtime starting on the third preoperative night and continuing throughout the third postoperative night
	Seet 2015 [47]	ASA I-II patients aged 21 to 65	Elective extraction of all four wisdom teeth under general anaesthesia	Melatonin 6 mg	36	Placebo	37	Oral premedication 90 min before surgery
	Khezri 2013 [35]	ASA I-III patients aged 25 to 80	Elective cataract surgery with intraocular lens implantation using phacoemulsification under topical anesthesia for the first time	Melatonin 3 mg	30	Placebo	30	Sublingual premedication 60 min before surgery
						Gabapentin 600 mg	40	
	Khezri 2013a [37]	ASA IV patients aged 35 to 85	Cataract surgery under retrobulbar nerve block	Melatonin 6 mg	40	Placebo	40	Sublingual premedication 90 min before arrival in the operating room
	Borazan 2010 [41]	ASA I-II patients aged 50 to 65	Elective open prostatectomy under general anesthesia	Melatonin 6 mg	26	Placebo	26	Oral premedication at the night before and 1 h before surgery
	Ismail 2009 [42]	ASA I–III patients older than 60 years	Cataract surgery with intraocular lens implantation under topical anesthesia	Melatonin 10 mg	20	Placebo	20	Oral premedication 90 min before surgery
	Caumo 2009 [44]	ASA I–II patients aged 19 to 60	Abdominal hysterectomy for myomatosis under regional anesthesia	Melatonin 5 mg	20	Placebo	20	Oral premedication the night before and 1 hour before surgery
						Clonidine 100 mg	19	
	Caumo 2007 [45]	ASA I-II aged 30 to 55	Abdominal hysterectomy under regional anesthesia	Melatonin 5 mg	17	Placebo	16	Oral premedication at the night before and 1 h before surgery
Inflammatory pain	de Zanette 2014 [34]	Females aged 18 to 65	Fibromyalgia	Melatonin 10 mg and amitriptyline 25 mg	21	Amitriptyline 25 mg	21	Oral premedication at bedtime for 6 weeks
				Melatonin 10 mg	21			
	Vidor 2013 [68]	Females aged 20 to 40	Myofascial temporomandibular disorder	Melatonin 5mg	16	Placebo	15	Oral premedication at bedtime for 4 weeks
	Schwertner 2013 [38]	Female patients aged 18 to 45	Endometriosis-associated chronic pelvic pain	Melatonin 10 mg	20	Placebo	20	Oral premedication at bedtime for 8 weeks
	Hussain 2011 [40]	Patients with primary fibromyalgia aged 18–65	Fibromyalgia syndrome	Melatonin 5 mg and placebo	27	Fluoxetine 20 mg and placebo	24	Oral premedication of melatonin as single daily dose at night time, and fluoxetine as single daily dose in the morning for 60 days.
				Melatonin 5 mg and fluoxetine 20 mg	23			
	Song 2005 [57]	Irritable bowel syndrome patients with sleep disturbances,aged 20 to 64	Abdominal pain induced by irritable bowel syndroms	Melatonin 3 mg	20	Placebo	20	Oral premedication at bedtime for 2 weeks
Procedural pain	Marseglia 2015 [67]	Children aged 1 to 14	Pain undergoing blood withdrawal	Melatonin 0.5 mg/kg (max 5 mg)	30	Placebo	30	Oral premedication 30 min before blood drawing
	Gitto 2012 [39]	Newborns of 32 weeks gestation or less	Endotracheal intubation	Melatonin 10 mg/kg, standard pharmacological and nonpharmacological therapy	30	Standard pharmacological and nonpharmacological therapy	30	Intravenously before endotracheal intubation
	Mowafi 2008 [43]	ASA I–II patients	Pain after intravenous cannula were placed under tourniquet	Melatonin 10 mg	20	Placebo	20	Oral premedication 90 min before surgery
Experimental pain	Andersen 2015 [30]	Healthy male volunteers aged 20 to 40	Validated burn injury	Melatonin 10 mg	29	Placebo	29	Intravenous administration 60 min before test
				Melatonin 100 mg	29			
	Stefani 2013 [36]	White healthy volunteers aged 19 to 47	Pressure and heat pain	Melatonin 0.05 mg/kg	15	placebo	15	Sublingual premedication 30 min before test
				Melatonin 0.15 mg/kg	15			
				Melatonin 0.25 mg/kg	16			

**Figure 3:**
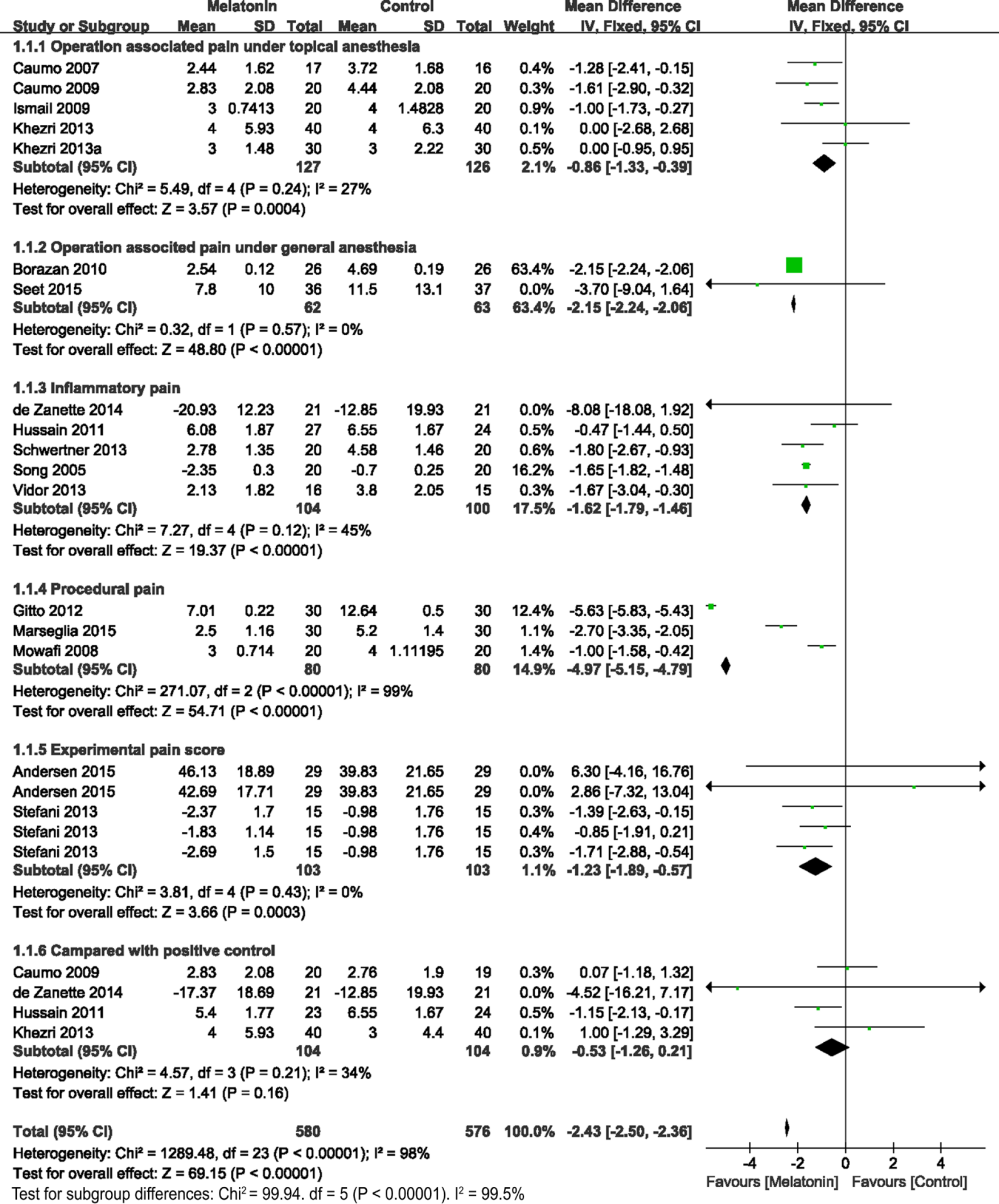
Forest plot: pain intensity indicated by the pain score

Pain intensity indicated by pain scores was the primary outcome in this meta-analysis. This outcome has been reported in 18 studies. Figure 3 showed the significantly strong efficacy of melatonin over the control on the anti-nociception effect (WMD = –2.43, 95% CI, –2.5 to –2.36; *P* < 0.00001) with significant statistical heterogeneity (I^2^ = 98%). A subgroup analysis was conducted: compared with placebo, melatonin administration significantly decreased the scores of operation-associated pain under topical anesthesia (WMD = –0.86, 95% CI, –1.33 to –0.39; *P* = 0.0004; I^2^ = 27%), operation-associated pain under general anesthesia (WMD = –2.15, 95% CI, –2.24 to –2.06; *P* < 0.00001; I^2^ = 0%), inflammatory pain (WMD = –1.62, 95% CI, –1.79 to –1.46; *P* < 0.00001; I^2^ = 45%), procedural pain (WMD = –4.79, 95% CI, –5.15 to –4.79; *P* < 0.00001; I^2^ = 99%), and experimental pain (WMD = –1.23, 95% CI, –1.89 to –0.57; *P* = 0.0003; I^2^ = 0%). Significant heterogeneity for included subgroup differences was indicated (I^2^ = 99.5%, *P* < 0.00001). Among all of the subgroups, no statistical heterogeneity was indicated except for the procedural pain group. As shown in Table 2, the procedural pain group included pain under blood withdrawal, endotracheal intubation and intravenous cannula placement. Although significant anti-nociception effects of melatonin were shown in every study, the beneficial scales of melatonin were different among these procedural operations. Further subgroup analysis requires more trials. Additionally, melatonin was comparable to the positive control (WMD = –0.53, 95% CI, –1.26 to 0.21; *P* = 0.16; I^2^ = 34%). Sensitive analysis showed that no study significantly influenced the reliability of the pooled results.

**Figure 4:**
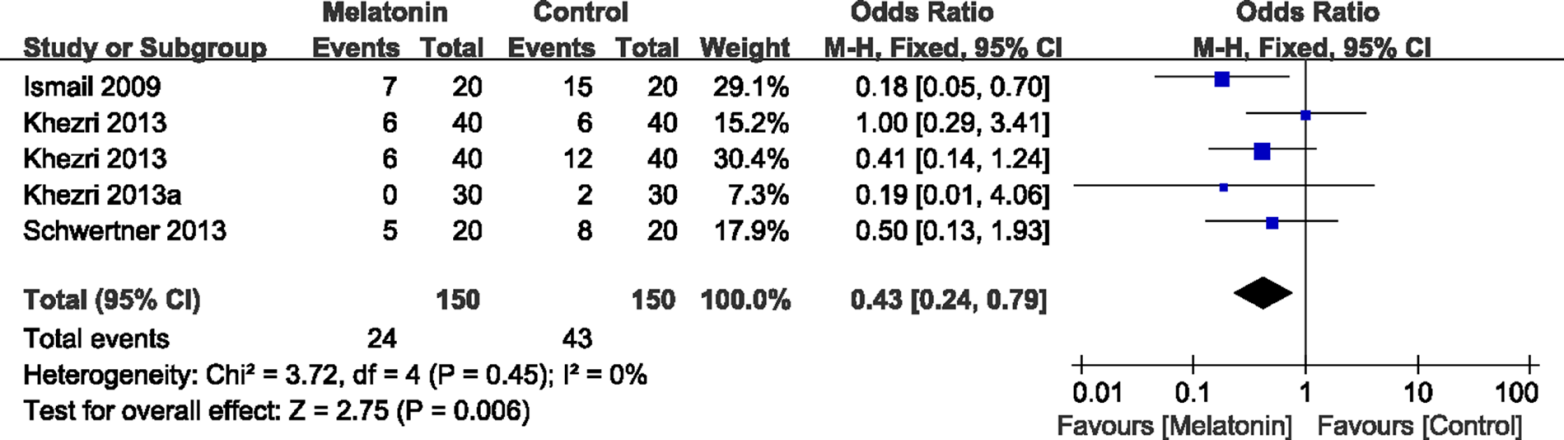
Forest plot: proportion of patients with analgesic requirements

**Figure 5:**
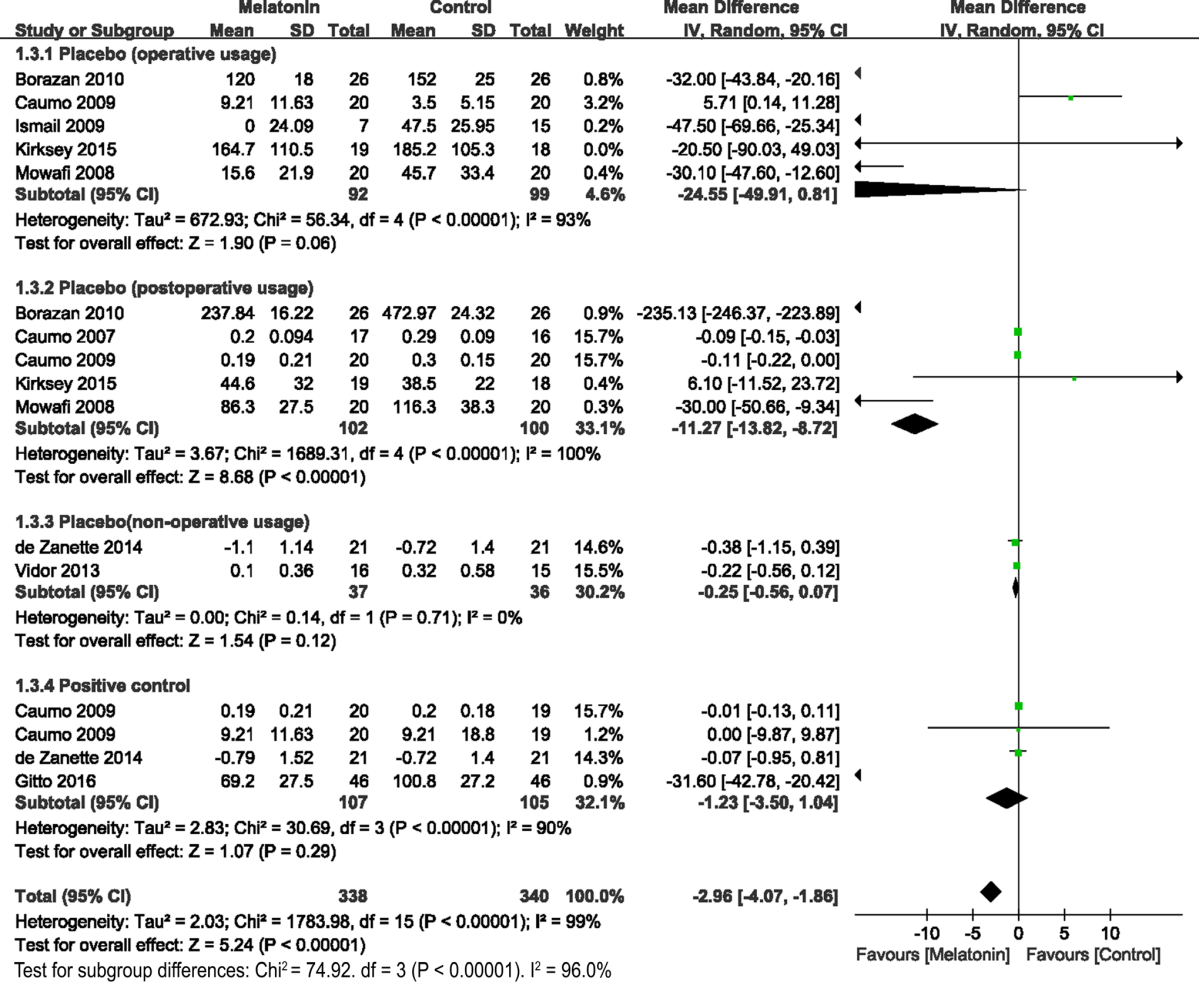
Forest plot: total analgesic consumption

**Figure 6:**
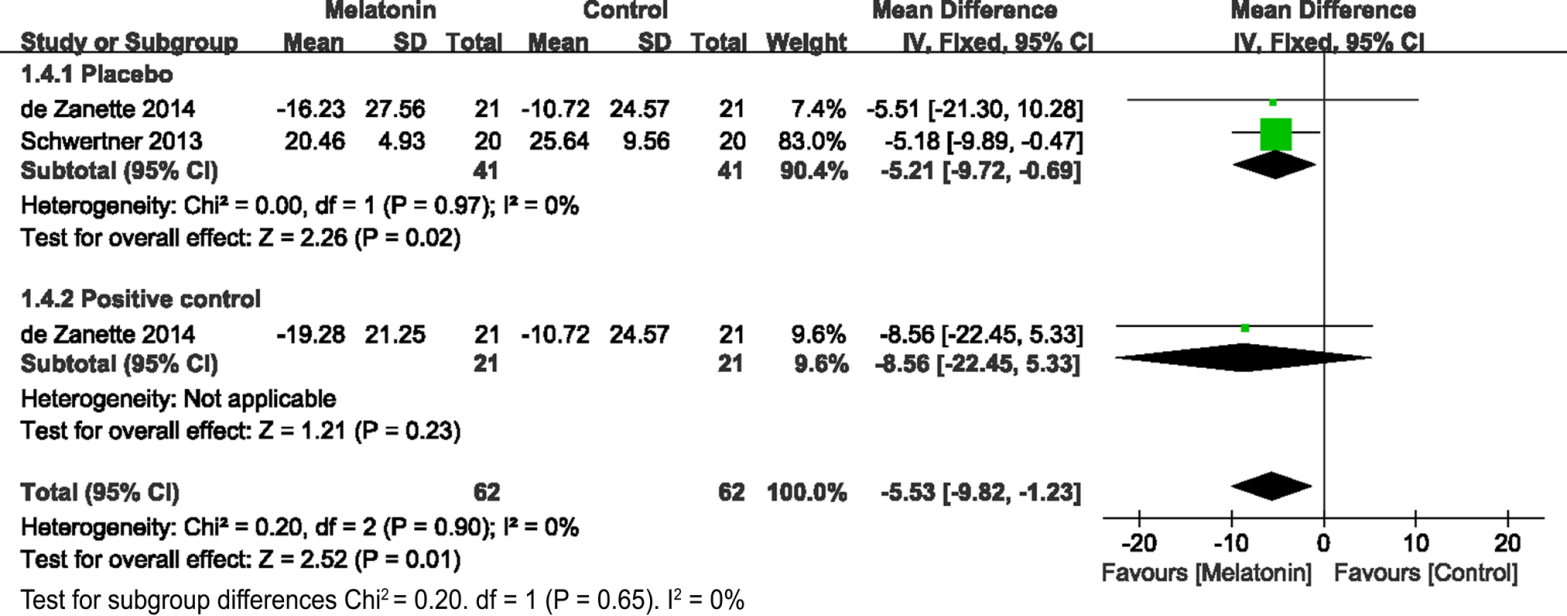
Forest plot: BDNF levels BDNF, brain-derived neurotrophic factor.

The secondary outcomes were the number of patients with analgesic requirements (Figure 4), total analgesic consumption (Figure 5), and BDNF levels (Figure 6). As reported in 4 studies, melatonin administration significantly decreased the proportion of patients requiring analgesic drugs (OR= 0.43, 95% CI, 0.24 to 0.79; *P* = 0.006; I^2^ = 0%) (Figure 4). A random-effects model was used to evaluate the effects of melatonin on requirements with analgesic drugs (Figure 5). Pooling data from 10 studies revealed significantly fewer patients with analgesic requirements in the melatonin group (Random model, WMD = –2.69, 95% CI, –4.07 to –1.86; *P* < 0.00001; I^2^ = 99%). Further subgroup analysis showed that melatonin significantly reduced the postoperative usage of analgesic drugs (Random model, WMD = –11.27, 95% CI, –13.82 to –8.72; *P* < 0.00001; I^2^ = 100%), while no significant reductions were observed in operative usage (Random model, WMD = –24.55, 95% CI, –49.91 to 0.81; *P* = 0.06; I^2^ = 93%) and non-operative usage (Random model, WMD = –0.25, 95% CI, –0.56 to 0.07; *P* = 0.12; I^2^ = 0%). However, melatonin was comparable to the positive control (Random model, WMD = –1.23, 95% CI, –3.50 to 1.04; *P* = 0.29; I^2^ = 90%). Sensitivity analysis found that after exclusion of results from Borazan et al in the postoperative group, the overall effects (Random model, WMD = –0.17, 95% CI, –3.50 to 1.04; *P* = 0.26; I^2^ = 86%) and effects in postoperative usage (Random model, WMD = –0.10, 95% CI, –0.27 to 0.07; *P* = 0.23; I^2^ = 65%) became insignificant. Furthermore, melatonin also significantly downregulated BDNF levels (WMD = –5.43, 95% CI, –22.45 to –1.23; *P* = 0.001; I^2^ = 0%) (Figure 6).

The funnel plot is shown in Figure 2. No significant publication bias was observed (Eggers test: 1.121428, 95% CI: –3.588125 to 5.830981, *P* = 0.623). No further trials were identified through extensive searches. No significant adverse events were reported in the included studies. Because the safety of exogenous melatonin supplementation has been confirmed, we did not pool the data relevant to this outcome.

## DISCUSSION

This pooling study showed that melatonin could significantly decrease the intensity of every type of pain, including operation-associated pain under topical anesthesia, operation-associated pain under general anesthesia, inflammatory pain, procedural pain, and experimental pain. The mechanism for this reduction may depend on decreasing BDNF levels. Furthermore, melatonin also decreased the proportion of patients requiring additional analgesic drugs, but its effects on total analgesic consumption still require further confirmation.

In the excluded studies, the analgesic effects of melatonin were also demonstrated. In critically ill patients, long-term administration of melatonin also significantly improved the patients’ pain [52]. Two other studies showed that melatonin significantly reduced the frequency and intensity of nocturnal pain in patients with ulcer-like dyspepsia [53] and the intensity of visceral pain in patients with irritable bowel syndrome [54]. Another trial was designed to investigate the efficacy of melatonin in Intensive Care Unit patients [55]. Accompanying the alleviation of pain, melatonin also significantly improved sedation and anxiety [35, 37, 42, 43]. Similarly, compared with midazolam, melatonin also significantly contributed to sedation induction in children [31]. These studies were excluded primarily due to their unavailable data about the included outcomes, especially the pain scores.

The diurnal variation of pain tolerance may be attributed to melatonin variation [21]. Further evidence has shown that endogenous elevation of melatonin also improves pain tolerance. Probiotic administration increased the rectal distension pain threshold in irritable bowel syndrome and elevates morning melatonin levels [56]. In particular, the increased morning melatonin levels were correlated with improved bowel habits and irritable bowel syndrome-associated pain (with a regression coefficient of 0.61) [56]. This study is coincident with the observation that direct supplement of melatonin also improved irritable bowel syndrome-associated visceral pain [54, 57].

Melatonin is involved in the physiopathology of pain. BDNF is an important mediator and a central modulator of pain [58–60]. In neuropathic pain, BDNF release has been observed in the spinal cord, contributing to chronic pain [61]. Further study found that BDNF contributed to hyperpathia through presynaptic GABAergic inhibition [62]. In the colonic mucosa of patients with irritable bowel syndrome, increased BDNF expression was correlated with visceral hyperalgesia and increased abdominal pain scores [63]. The mechanisms for these observations may be involved in elevating tyrosine receptor kinase B expression [63]. BDNF is an important regulator of pain, and the anti-nociceptive mechanisms of melatonin may be attributed to decreased BDNF levels [34]. Furthermore, production of inflammatory factors is important to induce and maintain pain [64]. Melatonin can also reduce inflammatory cytokine levels, including interleukin (IL)-6, IL-8, IL-10 and IL-12, in newborns undergoing intubation and mechanical ventilation [39]. Another experimental animal study showed that melatonin significantly attenuated inflammation-mediated hyperalgesia in rats [65].

The analgesic effects of melatonin may also be influenced by several factors, such as the duration of administration and gender. In an experimental animal study with Freund’s adjuvant-induced inflammatory pain, the different administration durations of melatonin differently influenced the BDNF levels [66]. To be specific, short-term (3 days), but not long-term (8 days) administration of melatonin increased BDNF levels [66]. The analgesic effects of melatonin may be better in females than in males [47]. Seet et al. [47] found insignificant effects of melatonin in patients undergoing elective extraction of all four wisdom teeth, while further subgroup analysis showed a positive effect of melatonin in female, but not male, patients. Sexual dimorphism has also been indicated in other studies [56]. Elevated melatonin levels due to probiotics are observed in male patients, but not in female patients, further alleviating pain. In particular, the anti-nociceptive effects of melatonin in the pediatric population have been explored. However, further confirmation regarding melatonin application in this population is needed from more clinical trials.

In this meta-analysis, we conducted a systematic search for melatonin administration on anti-nociception. The pooling data included all available RCTs, involving 1053 patients. Strong evidence supports the utilization of melatonin on anti-nociception against many types of pain. Several limitations need to be considered. First, profound heterogeneity of the secondary outcome was found, i.e., total analgesic consumption. Although further subgroup analyses were performed to identify the heterogeneity source, no potential methodological and clinical sources of heterogeneity were identified. The considerable heterogeneity was also unaffected by exclusion of individual RCTs. Thus, a random-effects model was used for these data. Furthermore, the magnitude of this outcome was influenced by some studies. Second, although the data analysis demonstrated that melatonin significantly reduced the proportion of patients with analgesic requirements and decreased BDNF levels, these results may need support from more studies. Third, several influencing factors, such as duration of administration and sexual dimorphism in melatonin’s analgesic effects, have been indicated; however, these observations were limited by the original design of the clinical trials and need to be further validated in well-designed studies.
